# Using Sensitivity Analysis to Develop a Validated Computational Model of Post-operative Calvarial Growth in Sagittal Craniosynostosis

**DOI:** 10.3389/fcell.2021.621249

**Published:** 2021-05-26

**Authors:** Connor Cross, Roman H. Khonsari, Leila Galiay, Giovanna Patermoster, David Johnson, Yiannis Ventikos, Mehran Moazen

**Affiliations:** ^1^Department of Mechanical Engineering, University College London, London, United Kingdom; ^2^Service de Chirurgie Maxillo-Faciale et Plastique, Assistance Publique des Hôpitaux de Paris, Paris, France; ^3^Department of Neurosurgery, Craniofacial 16 Surgery Unit, Necker–Enfants Malades University Hospital, Assistance Publique–Hôpitaux de 17 Paris, Université de Paris, Paris, France; ^4^Oxford Craniofacial Unit, Oxford University Hospital, NHS Foundation Trust, Oxford, United Kingdom

**Keywords:** craniosynostosis, cerebrospinal fluid, finite element, calvarial growth, sagittal synostosis, biomechanics

## Abstract

Craniosynostosis is the premature fusion of one or more sutures across the calvaria, resulting in morphological and health complications that require invasive corrective surgery. Finite element (FE) method is a powerful tool that can aid with preoperative planning and post-operative predictions of craniosynostosis outcomes. However, input factors can influence the prediction of skull growth and the pressure on the growing brain using this approach. Therefore, the aim of this study was to carry out a series of sensitivity studies to understand the effect of various input parameters on predicting the skull morphology of a sagittal synostosis patient post-operatively. Preoperative CT images of a 4-month old patient were used to develop a 3D model of the skull, in which calvarial bones, sutures, cerebrospinal fluid (CSF), and brain were segmented. Calvarial reconstructive surgery was virtually modeled and two intracranial content scenarios labeled “CSF present” and “CSF absent,” were then developed. FE method was used to predict the calvarial morphology up to 76 months of age with intracranial volume-bone contact parameters being established across the models. Sensitivity tests with regards to the choice of material properties, methods of simulating bone formation and the rate of bone formation across the sutures were undertaken. Results were compared to the *in vivo* data from the same patient. Sensitivity tests to the choice of various material properties highlighted that the defined elastic modulus for the craniotomies appears to have the greatest influence on the predicted overall skull morphology. The bone formation modeling approach across the sutures/craniotomies had a considerable impact on the level of contact pressure across the brain with minimum impact on the overall predicated morphology of the skull. Including the effect of CSF (based on the approach adopted here) displayed only a slight reduction in brain pressure outcomes. The sensitivity tests performed in this study set the foundation for future comparative studies using FE method to compare outcomes of different reconstruction techniques for the management of craniosynostosis.

## Introduction

The cranium consists of several bones that are connected via cranial joints or sutures. Sutures facilitate the birth and accommodate the radial expansion of the brain during infancy (Anatole and Dekaban, 1977; Morriss-Kay and Wilkie, 2005; Lieberman, 2011; Richtsmeier and Flaherty, 2013; Jin et al., 2016; Adigun and Al-Dhahir, 2017; Hegazy and Hegazy, 2018). Early fusion of the sutures is a medical condition called craniosynostosis with the most common form of this condition being the early fusion of the sagittal suture i.e., occurring in ca. 3 per 10,000 live births (Morriss-Kay and Wilkie, 2005; Cunningham and Heike, 2007; Johnson and Wilkie, 2011; Cornelissen et al., 2016; Kalantar-Hormozi et al., 2019). The condition results in limited expansion of the skull perpendicular to the fused suture, leading to compensatory anteroposterior growth. In addition, raised intracranial pressure may cause cognitive impairment and visual loss (Gault et al., 1992; Lo and Chen, 1999). Various calvarial reconstructions to alleviate and correct these abnormalities have existed since the late nineteenth century (Lane, 1892; Lauritzen et al., 2006; Rocco et al., 2012; Simpson et al., 2015; Mathijssen, 2015; Microvic et al., 2016) with their various cognitive and morphological outcomes debated and compared to optimize the management of this condition (Hashim et al., 2014; Isaac et al., 2018; Magge et al., 2019).

Finite element (FE) method is a powerful computational tool that has been widely used in the field of biomechanics for the design and development of various structures and systems. The same technique has huge potentials to optimize the management of various form of craniosynostosis (e.g., You et al., 2010; Wolański et al., 2013; Malde et al., 2019; Dolack et al., 2020). Several recent studies have developed validated computational model of calvarial growth in rodent (Lee et al., 2017; Marghoub et al., 2018), and human infant models (Libby et al., 2017; Weickenmeier et al., 2017) as well as predicting follow up results in treated sagittal craniosynostosis patients (Malde et al., 2020). However, few studies have carried out detail investigations to understand the sensitivity of these models to the choice of their input parameters (Barbelto-Andres et al., 2020). Such sensitivity studies are crucial to advance our understanding of the limitations of FE models as well as achieving more accurate predictions of the skull growth using this method.

The aim of this study was to carry out a series of sensitivity studies to understand the effect of various input parameters on predicting the skull morphology of a sagittal synostosis patient post-operatively. Therefore, a preoperative patient-specific finite element model was developed. The post-operative skull morphology and the level of contact pressure at the intracranial volume (ICV)-bone interface were quantified and compared across a number of sensitivity tests.

## Materials and Methods

### Patient Computed Tomography Data

**FIGURE 1:**
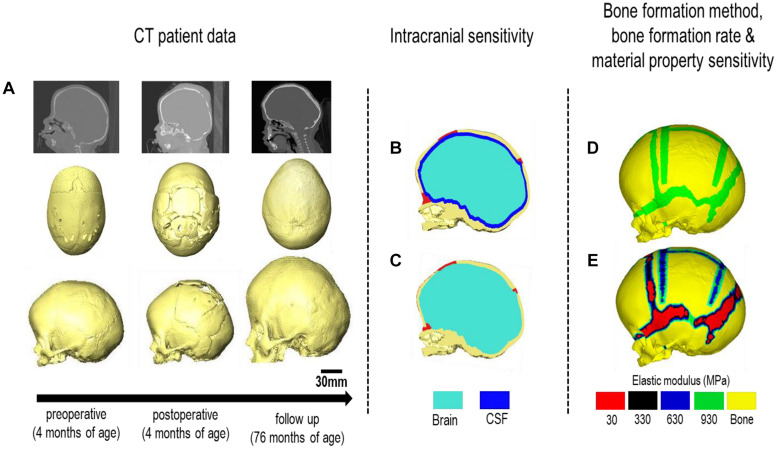
Workflow of the study. CT images of the patient are obtained at various treatment stages. **(A)** The preoperative CT is used for image processing and 3D reconstruction. Two intracranial volume models **(B,C)** were then compared under differing bone formation methods **(D,E)**.

Computed tomography (CT) images of a sagittal craniosynostosis patient were retrieved from the Hôpital—Necker Enfants—Malades Cranio-facial Surgery Unit (Paris, France) at a resolution of 0.625 × 0.625 mm. Full ethical consent from the center and the patients’ guardians was granted for the purposes of this study. Preoperative and immediate post-operative images were taken at 4 months of age and 6 days after the operation, respectively. Long term follow up CT images were taken at 76 months of age (i.e., 72 months after the operation). Anatomical 3D segmentation of the preoperative CT data was performed in Avizo image processing software (Thermo Fisher Scientific, Mass, United States). The follow up data at 76 months was used for morphological validation. 3D reconstructions of all CT data are highlighted in Figure 1A at each time point.

### Model Development

Segmentation of the calvarial bone, sutures, and the ICV was undertaken. The segmentation consisted of four components: (1) Calvarial bone (frontal, parietal, occipital, temporal, and craniofacial bones); (2) Sutures (metopic, squamosal, coronal, lambdoid, anterior fontanelle, frontozygomatic and zygomaticotemporal); (3) cerebrospinal fluid (CSF) and (4) the brain (frontal lobe, temporal lobe, parietal lobe, occipital lobe and cerebellum). Bone was segmented automatically based on grayscale values while other tissues were segmented manually. The mandible was removed from the segmentation as the primary focus was on calvarial growth.

The *in vivo* surgical craniotomies (i.e., Renier’s “H” technique) were also replicated across the calvaria (Rocco et al., 2012) and confirmed by the surgical team (i.e., Roman H. Khonsari and Giovanna Patermoster). A 3–4-cm wide rectangular cut was performed across the parietal, posterior of the coronal and anterior of the lambdoid sutures. The fused suture was removed and divided into two square portions. These were then reinserted to aid with long term calvarial healing. Two wedges extending from craniotomy-squamosal were created on each side of the parietal bone to assist with post-operative skull widening and anteroposterior shortening.

The ICV was modeled under two conditions (Figures 1B,C):

Model I: CSF present consisted of a uniform 2–3 mm thick material layer defined as CSF between the cranial bones and the brain. Due to the resolution of the CT images, accurate *in vivo* representation of the CSF could not be achieved. Therefore, the aforementioned thickness was used based on previous studies (see e.g., Lam et al., 2001; Clouchoux et al., 2012).
Model II: CSF absent defined the total ICV as the brain for comparison. Model II was used as the baseline approach for our sensitivity studies. Following segmentation, the surface model of the skull was transformed into a meshed solid geometry in Avizo that was then imported into a finite element package.

### Finite Element Analysis

Both models were imported into ANSYS finite element software (Canonsburg, United States) as solid meshed models. A quadratic tetrahedral mesh consisting of 3,100,000 elements across the skull and 900,000 elements across the CSF-brain was chosen after a mesh convergence analysis (i.e., several models were imported from Avizo to ANSYS in this respect). Correction of element intersection and poor aspect ratios was performed prior to importation. All material properties were defined as linear isotropic. For both models, the cranial bones, sutures and craniotomies were initially assigned a baseline elastic modulus of 3,000, 30, and 30 MPa, respectively (McPherson and Kriewall, 1980; Moazen et al., 2015—these were altered later—see sensitivity tests section). The brain (intracranial volume) elastic modulus was defined as 100 MPa (Libby et al., 2017) and the CSF elastic modulus was defined as 40 MPa. The Poisson’s ratio of the cranial bones, sutures and craniotomies was assumed to be 0.3. The Poisson’s ratio of CSF was assumed to be 0.48. Note, since the exact values/distribution of CSF pressure across the skull are not still clear, and modeling the CSF as fluid was beyond the scope of this study, we decided to model the impact of CSF on the prediction of calvarial growth and ICV surface pressures using solid elements.

### Boundary Conditions and Modeling of the Growth

A surface-to-surface penalty-based contact was established between the ICV and inner-calvarial interface for both models. These interfaces were initially in contact, after which normal and tangential friction behavior during calvarial growth was granted. A friction coefficient of 0.1, a penetration tolerance of 0.5, and a normal penalty stiffness of 600 N/mm was used at all interfaces where contact was defined. These values were chosen based on our previous sensitivity tests (Malde et al., 2020). A “bonded” interface behavior was enforced between bone, suture, craniotomies and CSF surfaces though out all simulations, i.e., allowing no relative motion at the aforementioned interfaces.

Nodal constraints in all degrees of freedom were placed around the foramen magnum and along the nasion to avoid rigid displacement during skull growth. The radial expansion of the brain/ICV was modeled using thermal analogy as described in detail elsewhere (see Libby et al., 2017; Marghoub et al., 2018, 2019; Malde et al., 2020). To summaries, a linear isotropic expansion was applied to the brain/ICV, where the pre-operative ICV (measured at 659 ml) was expanded to follow up ICV at 76 months of age (measured at 1,245 ml) in six intervals. The estimated age of each interval was calculated by measuring these new volumes (Sgouros et al., 1999). Two methods of bone formation were undertaken here:

Scenario I: applies a bone formation across the sutures/craniotomies as described in Marghoub et al. (2019) and here termed “gradual bone formation” (Figure 1D). Here, the suture and craniotomy elements within a specified radius from the adjacent bone were selected, at a rate of 0.1 mm for the sutures and 0.8 mm for the craniotomies for every month of volume growth (Mitchell et al., 2011; Thenier-Villa et al., 2018; Riahinezhad et al., 2019). To monitor for the level of strain in the selected elements, all elements with a hydrostatic strain (i.e., summation of all principal strains divided by three) within 0–50% were used. Scenario I was the baseline approach throughout the study.
Scenario II: here termed as “bulk bone formation” increased the bulk elastic modulus of the sutures/craniotomy as appose to simulating bone forming from the bone edge (Figure 1E). This method is computationally less expensive, i.e., solves faster but perhaps not as physiologically representative as the “gradual bone formation.” Further details are described by Malde et al. (2020).

### Sensitivity Tests

The baseline values as detailed above were changed using Model II under bone formation scenario I. Table 1 details respective sensitivity studies and their independent values, i.e., to the choice of material properties and rate of bone formation.

**TABLE 1:** Material property and bone formation rate sensitivity summary.

	Bone E (MPa), υ	Suture E (MPa), υ	Brain E (MPa), υ	Craniotomy E (MPa), υ	Suture formation rate (mm/month)
Baseline model	3,000, 0.3	30, 0.3	100, 0.48	30, 0.3	0.1
Test 1	421, 0.22	NA	NA	NA	NA
Test 2	NA	NA	NA	0.003, 0.3	NA
Test 3	NA	NA	0.003, 0.48	NA	NA
Test 4	NA	NA	NA	NA	0.2
Test 5	NA	NA	NA	NA	0.2 and 0.6 for suture and metopic/anterior fontanelle, respectively. Closure by 24 months.

**“E” refers to elastic modulus, “υ” refers to Poisson’s ratio and “NA” indicates no change from the Baseline model values.**

#### Material Properties

Three sensitivity analyses were performed to the changes in material properties.

Test 1–Bone Sensitivity: the elastic modulus of the bone was reduced from 3,000 to 421 MPa based on the previous study of Coats and Margulies (2006).
Test 2–Craniotomy Sensitivity: the elastic modulus of the craniotomies were reduced from 30 MPa to 3 kPa, i.e., two extremes that can capture wide range of tissues that can be present in these defects (see e.g., Leong and Morgan, 2008).
Test 3–Brain Sensitivity: the initial value of 100 MPa was reduced to 3 kPa based on nanoindentation studies performed on brain tissues (see e.g., Gefen et al., 2003).

#### Bone Formation Rate

This test further expanded on scenario II’s approach by altering the rate of bone formation across various sutures. This was carried out into two additional tests.

Test 4–Increased Formation Rate: here, we increased the original suture formation radius from 0.1 to 0.2 mm across all the sutures.
Test 5–Metopic and Anterior Fontanelle Closure: here, the complexity of test 4 was increased further. The bone formation rate across the metopic and anterior fontanelle was increased (i.e., 0.6 mm for each month) to replicate the early closure of these sutures. The metopic and the anterior fontanelle progressively closing from 4 months of age until closure is evident by 24 months (Pindrik et al., 2014; Teager et al., 2018). The rate specified for the bone formation across the craniotomy remained unchanged for both scenarios as specified in section “Boundary Conditions and Modeling of the Growth.”

#### Bone Formation Method and Effects of CSF

A comparison of both bone formation scenarios under both models was also undertaken to understand the effects our established CSF and various formation scenarios have on calvarial morphology and contact pressure outcomes across the ICV.

### Analysis

All simulations were subject to morphological comparison against the 76 months of age follow up CT data (see section “Patient Computed Tomography Data”) through a cross-sectional comparison and dimensional measurement of the length (from glabella to opisthocranion), width (between the left and right euryons) and height (from basion to bregma). All measurement and landmark placements were performed manually. The cephalic index (CI) was also calculated by multiplying the width against the height and dividing by 100. Bone formation rates were compared at various time points to establish the predicted sutures time of closure. A cross-sectional comparison and the level of contact pressure across the ICV was analyzed for both bone formation scenarios (Scenario I vs. II) under both models (Model I vs. II). Overall regional pressure across the ICV was measured to quantify areas of higher pressure.

## Results

### Material Properties

**FIGURE 2:**
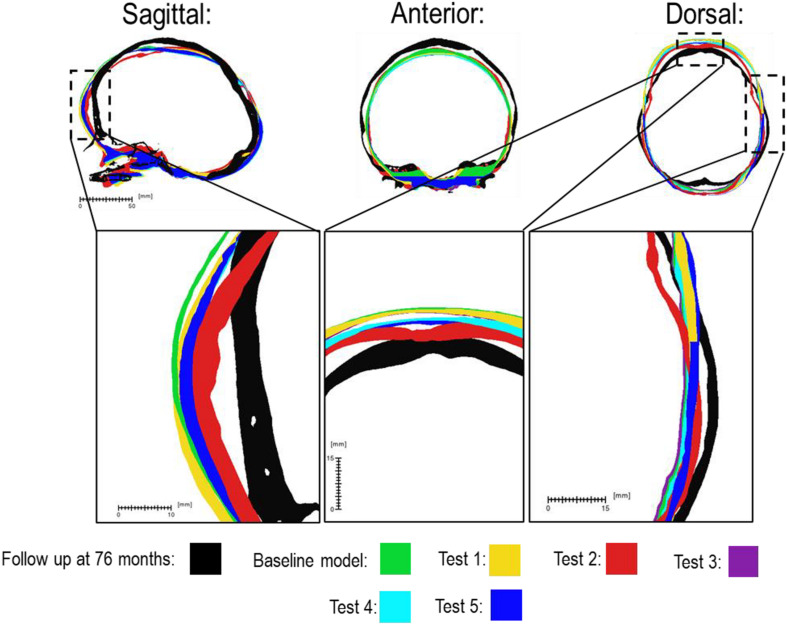
Material property and bone formation rate sensitivity cross-sections vs. follow up at 76 months of age. Dashed boxes indicate enhanced regions of interest.

There was a close match between all considered FE simulations (Test 1, 2, and 3) and the follow up CT skull morphology at 76 months of age (Figure 2). Minimal differences were observed across all material property sensitivities considered here, in terms of skull length, width and height measurements (Figure 2 and Table 2). Skull width and height measurements were lower than the follow up data while there was a close match between skull length measurement. Cephalic indexes of all considered sensitivity tests with respect to the changes in the material properties were in the range of 79.04–79.67 vs. the follow up CI of 86.62 (Table 2).

**TABLE 2:** Material property and bone formation rate sensitivity measurements.

	Length (mm)	Width (mm)	Height (mm)	Cephalic index
Baseline model	166.58	131.87	132.93	79.16
Test 1	166.9	132.97	132.43	79.67
Test 2	165.92	129.91	128	78.29
Test 3	166.52	131.62	132.87	79.04
Test 4	168.07	130.52	131.8	77.65
Test 5	169.56	131.3	131.57	77.43
Follow up at 76 months	166.17	143.94	137.23	86.62

### Bone Formation Rate

**FIGURE 3:**
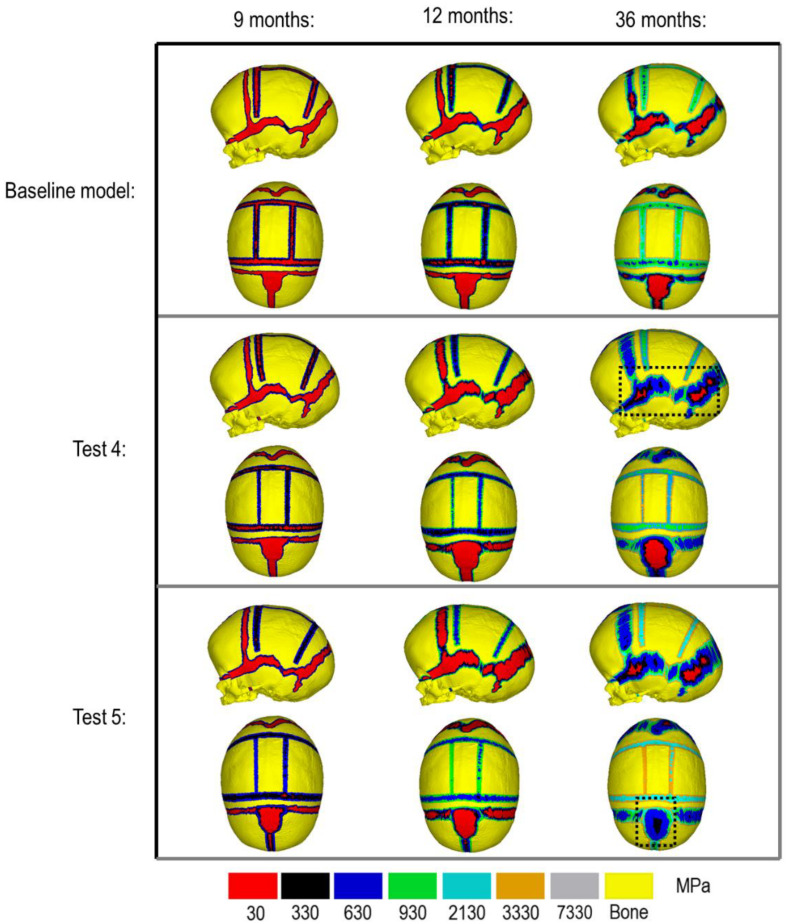
Bone formation rate sensitivity at various stages, sagittal and dorsal views.

Figure 3 compares the various bone formation rates (Baseline vs. Test 4 vs. 5) as detailed in section Sensitivity Tests. All outcomes predict the closure of the craniotomy by 12 months of age. The coronal suture displays complete closure between 36 and 76 months. The metopic, lambdoid, and squamosal regions remain marginally open, with various regions displaying closure. The anterior fontanelle remains open during the entirety of the growth cycle. Test 4 displays a near-complete closure of all sutures by 36 months of age, disregarding the anterior fontanelle which, similarly to the baseline comparison, remains open for the duration. All other sutures were found to have closed by the final 76 months of age interval. Test 5 displays an accelerated closure of the anterior fontanelle and metopic suture compared to the previous outcomes, which completely closes between 12 and 36 months of age. A close morphological match was seen against the follow up CT across all tests as seen in Figure 2.

### Bone Formation Method and Effects of CSF

**FIGURE 4:**
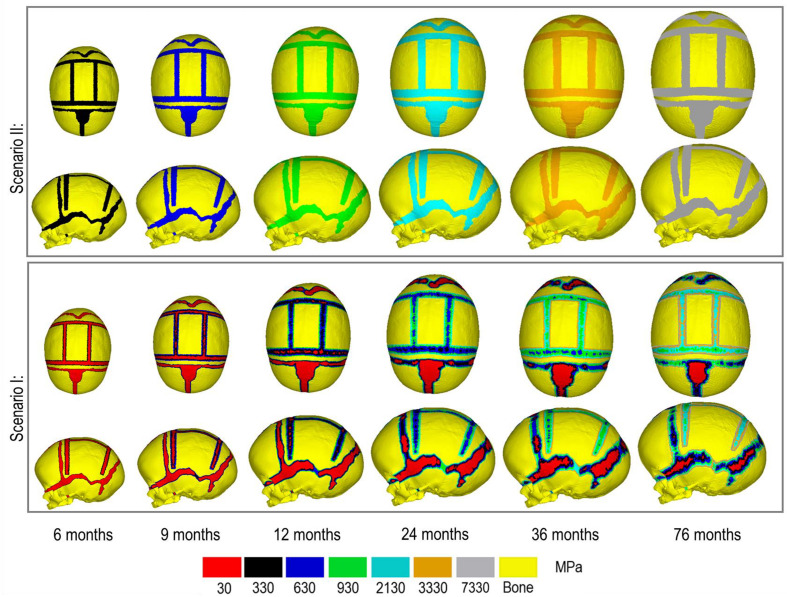
Bone formation methods under scenario I **(bottom)** and scenario II **(top)** during calvarial growth, sagittal and dorsal views (1:1 scale).

**FIGURE 5:**
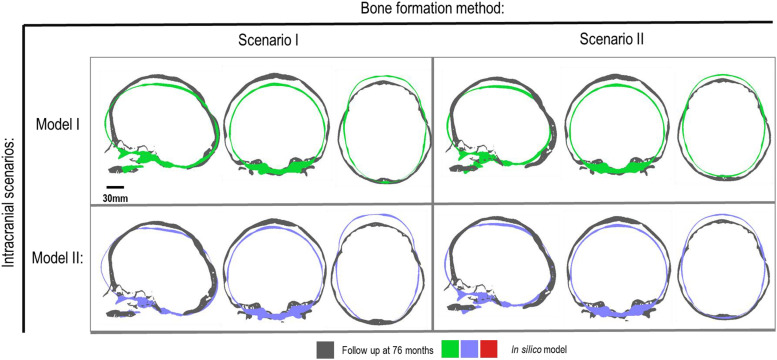
Cross-section analysis of intracranial scenarios (Model I and II) against both bone formation methods (Scenario I and II) at 76 months of age. Showing sagittal, anterior and dorsal planes.

Figure 4 represents the state of the various bone formation approaches at various ages. Figure 5 highlights the cross-sectional comparison of these bone formation approaches and the effects of CSF against follow up data with numerical measurements summarized in Table 3. Biparietal under-prediction and anterior over-prediction was observed in all outcomes. Model I approach (i.e., CSF present) does not appear to have any major implications to morphological outcomes when compared to Model II’s approach (i.e., CSF absent). Interestingly, despite the changes in modeling and formation method, there was no greatly varying impact on morphological outcomes, with all scenarios matching close to follow up data. This is further supported in the numerical measurements, where the length, width and height show an average of 159.9 mm, 129.7 mm and 129.1 mm, respectively. Cephalic index measurements ranged between 79.16 and 83.28 vs. follow up CI of 86.62 (Table 3).

**TABLE 3:** Summary of various Cerebrospinal fluid (CSF) model under each respective bone formation method.

Bone formation method:	Model	Length (mm)	Width (mm)	Height (mm)	Cephalic index
Scenario I	Model I	160.97	129.46	122.18	80.42
	Model II	166.58	131.87	132.93	79.16
Scenario II	Model I	155.86	129.81	128.31	83.28
	Model II	160.95	132.52	132.96	82.52
Follow up at 76 months		166.17	143.94	137.23	86.62

**FIGURE 6:**
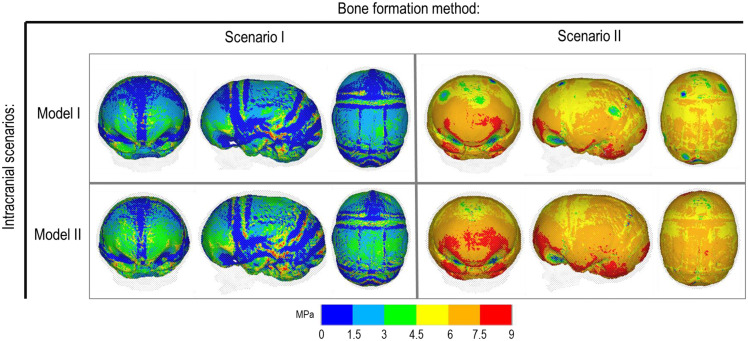
ICV pressure map at 76 months of age. Showing the dorsal, sagittal and anterior views.

Contact pressure mapping across the ICV surface is displayed in Figure 6, with the minimum, maximum and average pressure across each lobe region shown in Table 4. Incorporating CSF appears to only slightly reduce the average pressure across all regions. This is further supported by numerical outcomes, where the mean values do not vary more than 1 MPa between all scenarios. The chosen method of bone formation appears to have a greater role in contact pressure outcomes than the intracranial content chosen, where the average pressure across all lobes doubles, with the frontal and occipital lobe displaying the greatest difference (4.21–4.33 MPa and 4.49–4.52 MPa, respectively). A change that is also evident across the represented contact pressure maps.

**TABLE 4:** Summary of intracranial contact pressure outcomes across each region of interest.

		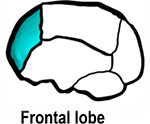			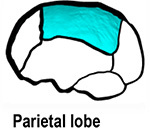			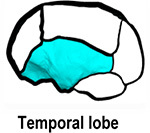			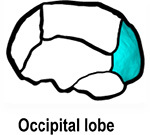			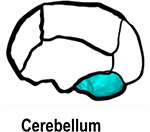		
Bone formation method:	Model	Min	Max	Mean	Min	Max	Mean	Min	Max	Mean	Min	Max	Mean	Min	Max	Mean
Scenario I	Model I	0	17.36	1.65	0	20.82	2.19	0	20.61	1.83	0	22.94	1.45	0	17.49	2.17
	Model II	0	15.40	2.29	0	18.62	2.68	0	22.63	2.33	0	16.95	1.87	0	20.46	2.86
Scenario II	Model I	0	8.83	5.98	0	10.97	5.69	0	18.55	5.66	0	11.37	5.94	0	11.61	6.64
	Model II	0	18.58	6.41	0	23.28	6.08	0	20.82	6.06	0	31.57	6.39	0	31.98	6.88

**Values are in MPa.**

## Discussion

There is a growing body of computational studies based on finite element method that are using this approach to optimize the clinical management of craniosynostosis. To the best of our knowledge, a few studies have carried out detailed sensitivity analysis to the choice of input parameters on the outcome of these models. In this study we investigated the impact of several key parameters on the outcome of a FE model, predicting calvarial growth in a patient-specific sagittal synostosis case. The identified parameters were changes in limited scenarios, based on what is perceived to be a reasonable estimate of their *in vivo* values based on the data in the literature, rather than a wide range of values for each parameter. Our results highlighted that pending the output parameter of interest (i.e., overall skull morphology after surgery or impact of surgical technique on the ICV pressure) the choice of input parameters can have a limited to major impact on the outcomes.

Considering the material property sensitivity tests performed here, our measurements showed the choice of craniotomies elastic modulus has the largest reduction on length (165.9 mm), width (129.9 mm) and height (128 mm) out of all the analyzed parameters (Table 2). Clinically craniotomies are gaps with “no material” present at these gaps post-operatively unless a medical device such as plates or springs are used. In the modeling approach implemented here, craniotomies were virtually assumed to be a “material” with low elastic modulus (i.e., low resistance to the applied forces). This approach allows us to model bone formation across the craniotomies that occur post-operatively. While a relatively low baseline elastic modulus was used in the initial models (i.e., 30 MPa similar to the suture properties and 100 times lower than the bone), our cross-sectional results highlight that the predicted skull morphology can be highly sensitive to this choice (Figure 2; red outline). This can be explained by the fact that the large displacements occur during the brain/ICV radial expansion across the craniotomies. Clinically (i) considering the operation modeled in this study, this closely replicates the purpose of these bitemporal craniotomies, which aims to increase the displacement of the bone mediolaterally while reducing anterior-posterior length (Rocco et al., 2012); (ii) this highlights that perhaps the number, position and orientation of craniotomies all contribute to the overall long term morphological outcomes of the surgery and variations observed.

During the natural development and following the surgical operation on craniosynostotic skulls, radial expansion of the skull occur hand in hand with bone formation across the sutures and craniotomies (Richtsmeier and Flaherty, 2013). We recently described a validated finite element-based approach to model the aforementioned phenomena in mice (Marghoub et al., 2019). In the present study for the first time, we applied the same methodologies to model the calvarial growth following calvarial reconstruction. A key unknown in translating our methodology from mouse to human was the rate of bone formation in the human, hence, the sensitivity tests to this choice were performed in this study. Our results highlighted that this parameter does not have a major impact on the overall predicted morphology of the skull (see Figure 2 for light and dark blue outlines). Gradually increasing the elastic modulus of the whole sutures/craniotomies sections under scenario II (i.e., “bulk bone formation”) also led to a close match between the overall predicted morphology of the skull and the *in vivo* data (see Figure 5 and also Malde et al., 2020). However, the rate of bone formation (Test 4 and 5) has an impact on the predicted pattern and timing (age) of sutures and craniotomies closure (see Figure 3 e.g., for highlighted dash lines across the anterior fontanels). Studies observing calvarial CT imaging and measurements observe that the majority of the sutures close between 30 and 40 months of age while small gaps might be present at most of the sutures except the metopic throughout life (Opperman, 2000; Lottering et al., 2016). In fact, the metopic and anterior fontanelle are suggested to fuse as early as 9 months of age (Hugh et al., 2001; Boran et al., 2018). Due to the lack of regular CT data for the patient considered here (that clinically is unethical to perform), detail validation of our predictions is challenging while overall it appears that regardless of the rate of bone formation the overall pattern of suture closures is similar to the *in vivo* data. With regards to the craniotomies, all comparisons present a complete closure by 9 months post-operative (12 months of age). This appears to match well with reported *in vivo* literature (e.g., Thenier-Villa et al., 2018). An important consideration when varying surgical techniques in which calvarial healing may prolong, which has been found to vary between different age groups and surgical methods (Hassanein et al., 2011; Thenier-Villa et al., 2018).

An alternative approach to the gradual bone formation approach described above is the “bulk bone formation.” The latter is computationally far less expensive and can model the changes in the overall stiffness of the sutures and craniotomies during the development or after surgery. This approach was used in our recent patient-specific modeling of calvarial growth (Malde et al., 2020). Our results here show that both methodologies can reasonably predict the overall morphology of the skull, however, these approaches lead to different levels of contact pressure across the brain/ICV. The gradual bone formation approach (i.e., scenario I) led to a lower level of contact pressure across the brain/ICV in comparison to the “bulk bone formation” (i.e., Scenario II) approach (see Figure 6 and Table 4). Another important parameter that can alter the predicted patterns of contact pressure across the brain is the CSF. CSF was modeled here using solid elements with low elastic modulus (see Supplementary Data for sensitivity tests to the impact of CSF elastic modulus on the contact pressure on the ICV). *In vivo*, CSF is obviously a fluid that plays a crucial role in nutrient transfusion across the brain with varied pressure during the development (see e.g., Moazen et al., 2016). Modeling the fluid-solid interaction at this interface was beyond the scope of this work. Yet, the sensitivity analysis performed here, considering its limitations, highlighted that CSF perhaps plays a smaller role on the level of contact pressure across the brain compared to the methods of bone formation during the calvarial growth/healing. Obviously, *in vivo* obstruction of CSF can lead to raised intracranial pressure with potential impacts on the brain that given the approach that was implemented here can be predicted by investigating the level of strain across the modeled CSF elements. Nonetheless, it may prove highly informative to investigate the contact pressures across different surgical techniques for the management of craniosynostosis and to correlate such results to the cognitive data (e.g., Chieffo et al., 2010; Hashim et al., 2014; Bellew and Chumas, 2015) to optimize management of craniosynostosis.

Perhaps the key limitations of the FE models and sensitivity tests described here are that: (1) the pattern of contact pressures on the brain/ICV was not validated and despite the efforts put into this work on including the effect of CSF further studies are required to advance our understanding of the *in vivo* level of loading at this interface; (2) the pattern of tissue differentiation across the sutures/craniotomies were not validated as such studies in human can be challenging. Nonetheless, given our previous studies in mice (e.g., Moazen et al., 2015), these predictions could be within the range of *in vivo* data; (3) bone was modeled as linear elastic homogenous material despite wide literature highlighting its anisotropy, variation in density, elastic modulus and mineral heterogeneity (e.g., Renders et al., 2008). Nonetheless given that at early stages of development and following calvarial reconstructions major deformations occur at the sutures and craniotomies perhaps this assumption could be acceptable or at least based on our results here it seems to have a minimal impact on predictions of calvarial growth in the age range and considering the treatment that was modeled here; (4) there are still differences between the predicted morphology at 76 month and the *in vivo* data (see differences between the outlines shown in Figures 2, 5) that can be e.g., due to manual deformation of the bones during the surgery that were not modeled in this study or the fact that our current modeling approach does not model facial growth that occur hand in hand with calvarial growth. Nonetheless, given the large deformation that the model has predicted i.e., about 72 months of growth considering all its limitations we think this a valuable model and approach that can be used in optimizing treatment of craniosysnotosis while advancing the methodologies implemented here.

In summary, the present study highlights how variations in material property, intracranial content, bone formation methods, and various bone formation rates may affect outcomes in predicting sagittal craniosynostosis correction. The discussed factors provided in this study lays the foundation to simulate various surgical reconstructions and observing their outcomes in correcting sagittal craniosynostosis.

## Data Availability Statement

The raw data supporting the conclusions of this article will be made available by the authors, without undue reservation.

## Ethics Statement

The studies involving human participants were reviewed and approved by the Necker–Enfants Malades University Hospital in Paris study No. 2018RK18. Written informed consent to participate in this study was provided by the participants’ legal guardian/next of kin.

## Author Contributions

CC performed the simulations and prepared the results. RK, LG, GP, and DJ provided the clinical case and advised on the clinical aspects of the study. MM, DJ, YV, RK, and GP designed the study. All authors contributed to the analysis of the data and preparation of the manuscript.

## Conflict of Interest

The authors declare that the research was conducted in the absence of any commercial or financial relationships that could be construed as a potential conflict of interest.

## Supplementary Material

Supplementary Figure 1 CSF elastic modulus sensitivity, contact pressure across the brain/ICV.
