# Supplementary material for: Using Sensitivity Analysis to Develop a Validated Computational Model of Post-operative Calvarial Growth in Sagittal Craniosynostosis
Source: Front Cell Dev Biol. 2021 May 26;9:621249. doi: 10.3389/fcell.2021.621249 (PMC8187911; doi:10.3389/fcell.2021.621249)

Supplementary figures

Supplementary 1: CSF elastic modulus sensitivity, contact pressure across the brain/ICV.

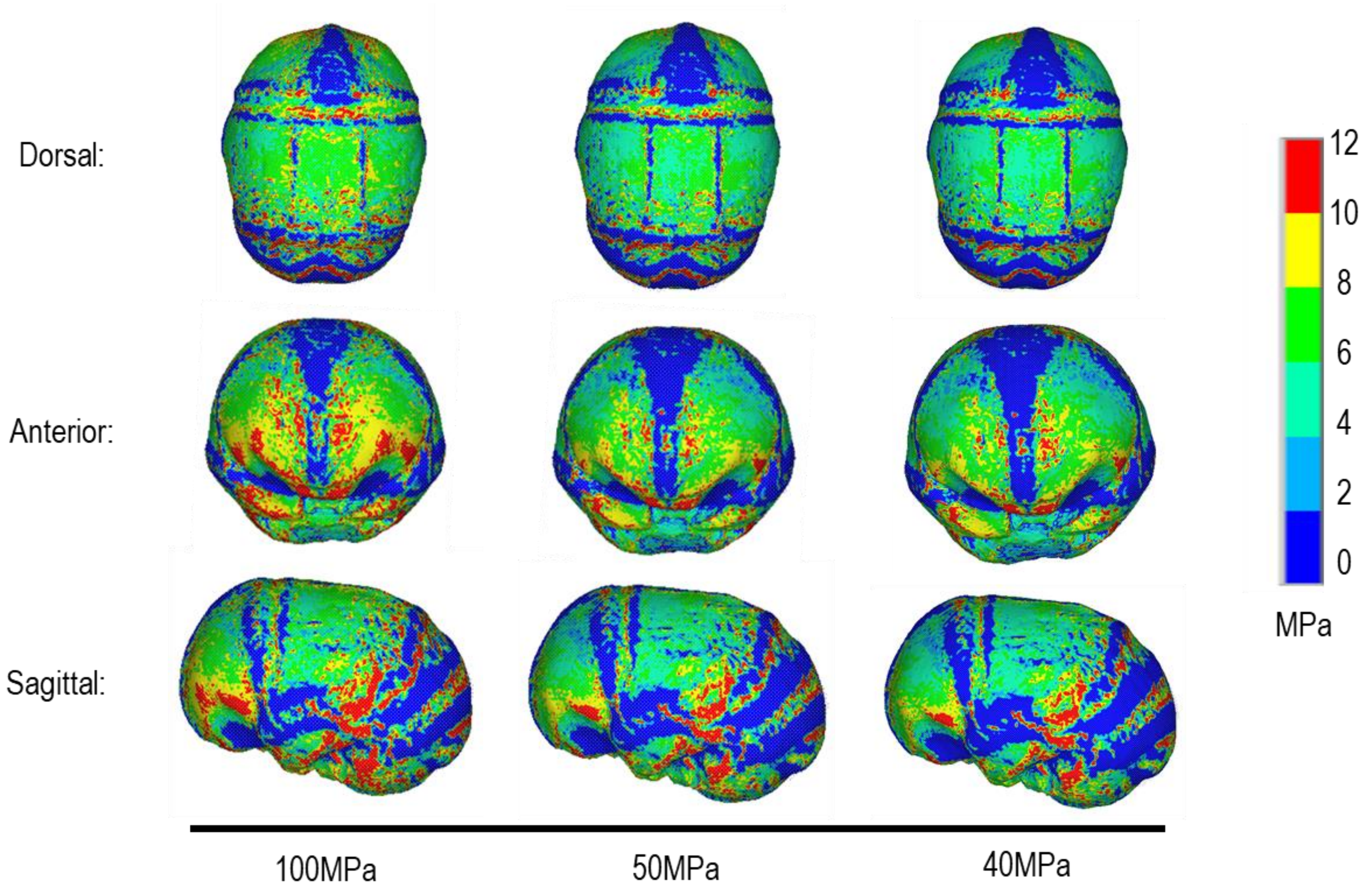

Supplement: Supplementary Figure 1 — CSF elastic modulus sensitivity, contact pressure across the brain/ICV. [file Image_1.pdf]
